# Bloom syndrome complex promotes FANCM recruitment to stalled replication forks and facilitates both repair and traverse of DNA interstrand crosslinks

**DOI:** 10.1038/celldisc.2016.47

**Published:** 2016-12-20

**Authors:** Chen Ling, Jing Huang, Zhijiang Yan, Yongjiang Li, Mioko Ohzeki, Masamichi Ishiai, Dongyi Xu, Minoru Takata, Michael Seidman, Weidong Wang

**Affiliations:** 1 Lab of Genetics, National Institute on Aging, National Institute of Health, Baltimore, MD, USA; 2 Lab of Molecular Gerontology, National Institute on Aging, National Institute of Health, Baltimore, MD, USA; 3 Laboratory of DNA Damage Signaling, Department of Late Effects Studies, Radiation Biology Center, Kyoto University, Kyoto, Japan

**Keywords:** BLM, Bloom syndrome, FANCM, Fanconi anemia, interstrand crosslinks, replication, RMI2

## Abstract

The recruitment of FANCM, a conserved DNA translocase and key component of several DNA repair protein complexes, to replication forks stalled by DNA interstrand crosslinks (ICLs) is a step upstream of the Fanconi anemia (FA) repair and replication traverse pathways of ICLs. However, detection of the FANCM recruitment has been technically challenging so that its mechanism remains exclusive. Here, we successfully observed recruitment of FANCM at stalled forks using a newly developed protocol. We report that the FANCM recruitment depends upon its intrinsic DNA translocase activity, and its DNA-binding partner FAAP24. Moreover, it is dependent on the replication checkpoint kinase, ATR; but is independent of the FA core and FANCD2–FANCI complexes, two essential components of the FA pathway, indicating that the FANCM recruitment occurs downstream of ATR but upstream of the FA pathway. Interestingly, the recruitment of FANCM requires its direct interaction with Bloom syndrome complex composed of BLM helicase, Topoisomerase 3α, RMI1 and RMI2; as well as the helicase activity of BLM. We further show that the FANCM–BLM complex interaction is critical for replication stress-induced FANCM hyperphosphorylation, for normal activation of the FA pathway in response to ICLs, and for efficient traverse of ICLs by the replication machinery. Epistasis studies demonstrate that FANCM and BLM work in the same pathway to promote replication traverse of ICLs. We conclude that FANCM and BLM complex work together at stalled forks to promote both FA repair and replication traverse pathways of ICLs.

## Introduction

Bloom syndrome (BS) and Fanconi anemia (FA) are two rare genetic diseases sharing several features, such as genomic instability, cancer predisposition and developmental abnormalities [1–3]. In addition, each disease has its own characteristics. For example, the cells from BS patients display a higher frequency of sister-chromatid exchanges (SCEs), which can lead to the loss of heterozygosity and increased cancer risks. Conversely, the cells from FA patients exhibit cellular hypersensitivity to drugs that induce DNA interstand crosslinks (ICLs), which can block essential DNA metabolic processes such as replication.

BS is caused by mutations in *BLM* gene, which belongs to the RecQ DNA helicase family conserved from *Escherichia coli* to humans [4]. In addition to BLM, two other human RecQ helicases are also mutated in the genomic instability diseases, Werner Syndrome [5] and Rothmund–Thomson syndrome, respectively [6], highlighting the essentiality of these enzymes in protecting genome integrity. BLM has been purified as a part of the DNA double Holliday junction dissolvasome complex that contains BLM, topoisomerase 3a (Top3a), RMI1 and RMI2 [7–10]. The four components of this complex work coordinately to catalyze dissolution of double Holliday junctions, which are intermediates produced during the repair of DNA double-strand breaks. This leads to suppression of crossover recombination and SCEs [11]. BLM is also recruited to stalled replication forks and is required for efficient recovery of the stalled forks [12–15].

Unlike BS that is caused by mutations in a single gene, at least 20 genes (*FANC-A*, *B*, *C*, *D1*, *D2*, *E*, *F*, *G*, *I*, *J*, *L*, *M*, *N*, *O*, *P*, *Q*, *R*, *S*, *T* and *U*) have been identified in which mutations can cause FA [1, 2, 16, 17]. The *FANC* gene products have been shown to act at various steps in the FA DNA damage response pathway to repair ICLs. Acting upstream of this pathway is the FA core complex that contains eight FA proteins (FANC-A, B, C, E, F, G, L and M) and five FA-associated proteins (FAAP100, FAAP24, FAAP20, MHF1 and MHF2) [18–28]. The main function of this complex is to monoubiquitinate the FA FANCI–FANCD2 complex (abbreviated as ID complex) in response to DNA damage and replication stress [29]. The ubiquitinated FA ID complex then recruits downstream FA proteins, as well as other repair molecules, to remove ICLs and restore stalled replication forks. FANCM and its dsDNA binding partner, MHF1 and MHF2, also constitute an independent complex, FANCM–MHF, which is conserved from yeast to human [23, 24]. This complex acts in a replication traverse pathway that enables the replication machinery to restart past the ICLs and complete the essential process of DNA synthesis at the expense of leaving the ICLs unrepaired [30]. These residual ICLs will be subsequently removed by post-replication repair mechanisms.

FANCM is a key component of both the FA core and FANCM–MHF complexes, and possesses critical DNA processing activities and functions [19, 23, 24, 31–35]. First, FANCM has specific binding activity for branched DNA structures, such as forks and Holliday Junctions; and this binding activity is required for recruiting FA core complex to damaged DNA and for monoubiquitination of the FA ID complex [23, 32, 33, 36, 37]. Second, FANCM harbors an ATP-dependent translocase activity that can remodel forks and Holliday junctions [19, 32, 33]. This activity is required for recovery of stalled replication forks [38–40], for activation of ATR kinase in response to replication stress [34, 40, 41], for cellular resistance to ICLs [33, 36, 37] and for replication traverse of ICLs [30]; but is dispensable for monoubiquitination of FANCD2 [33, 36, 37]. Third, FANCM contains multiple protein-interaction motifs and serves as a scaffold for assembly by MHF, FAAP24, the FA core complex, BS complex and PCNA [19, 22–24, 35, 42]. Mutations in FANCM that eliminate its interactions with its partners can disrupt the FA pathway, the replication traverse pathway, cellular resistance to ICLs, and/or suppression of SCEs [35, 42–44].

Structural analyses have shown that the interface between FANCM and the BLM complex consists of residues from MM2 motif of FANCM, as well as residues from RMI1 and RMI2 [43]. Mutations that disrupt this interface result in increased cellular sensitivity to ICLs, defective recruitment of BLM to stalled replication forks and a higher frequency of SCEs [35, 43]. However, the mechanism by which FANCM and BLM complex work together remains incompletely understood. Here we used chicken DT40 cells as a model to demonstrate that the interaction between FANCM and BLM complex is required for ATR-dependent recruitment of FANCM to stalled replication forks, for replication stress-induced FANCM phosphorylation and FA pathway activation, and for replication traverse of ICLs. Moreover, the helicase activity of BLM is important for FANCM recruitment to stalled forks and for the replication traverse of ICLs. Our data suggest that coordinated interactions between FANCM and BLM complex are necessary for their joint recruitment to stalled forks to promote both repair and traverse pathways of ICLs.

## Results

### FANCM co-localizes with BLM and FANCD2 at stalled replication forks

**Figure 1:**
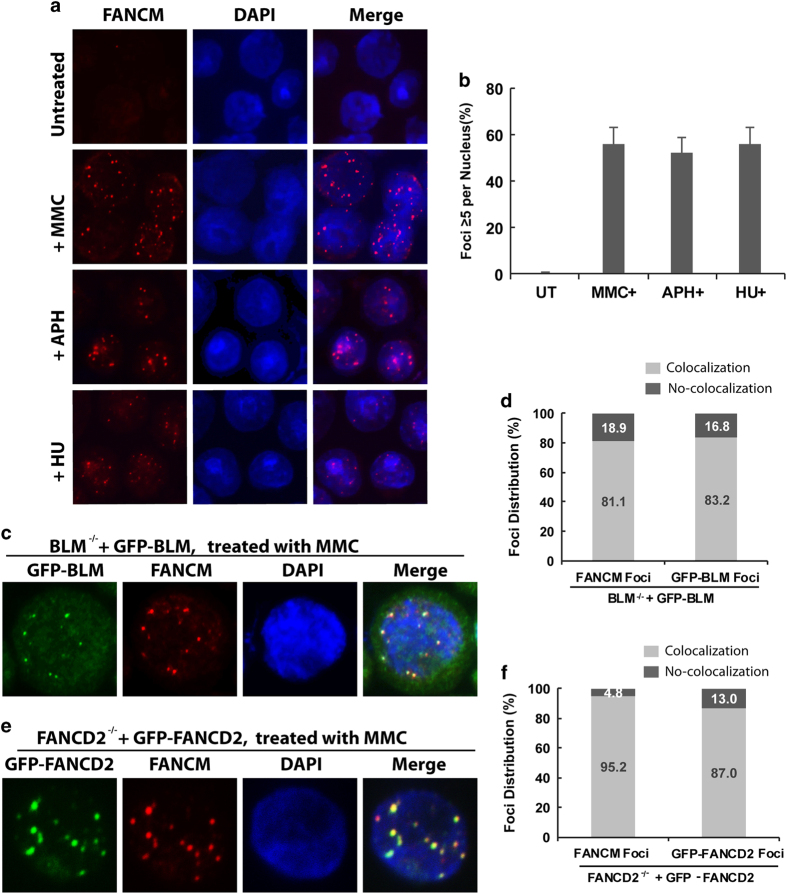
FANCM is recruited to stalled replication forks where it co-localizes with BLM and FANCD2. (**a**) Representative immunofluorescence images, and (**b**) their quantification, show that FANCM foci were induced in DT40 cells treated by MMC, APH and HU, respectively. The cells were either untreated (UT), or treated with MMC (60 ng ml^−1^), APH (1.25 μg ml^−1^) or HU (1.5 μm) for 18 h; and then were assayed for FANCM focus formation. The mean and standard deviations (s.d.) of the percentage of cells containing ⩾5 FANCM foci were shown in the graph. (**c**) Immunofluorescence images and (**d**) quantification show that majority of MMC-induced FANCM foci are co-localized with GFP-tagged BLM foci. *BLM*^*−/−*^ DT40 cells stably expressing GFP-tagged BLM were treated with MMC (60 ng ml^−1^) for 18 h. The graph in **d** shows the percentages of FANCM and GFP foci that have either co-localization or no co-localization, as assayed in **c**. (**e**, **f**) The same as described in **c**, **d**, respectively; except that *FANCD2*^*−/−*^ DT40 cells stably expressing GFP-tagged FANCD2 were used. APH, aphidicolin; GFP, green fluorescence protein; HU, hydroxyurea; MMC, mitomycin C.

**Figure 2:**
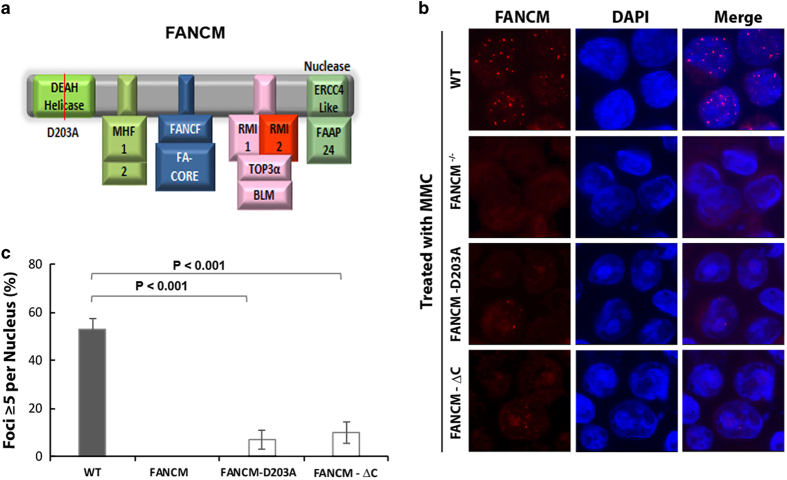
The recruitment of FANCM to stalled forks requires its translocase activity and interaction with FAAP24. (**a**) A schematic diagram depicting the FANCM helicase domain and protein-interaction motifs for its binding partners. (**b**) Representative immunofluorescence images and (**c**) their quantification show that the MMC-induced focus formation of FANCM is reduced by mutations in the FANCM helicase domain and the C-terminal FAAP24-interaction domain [36]. Various DT40 cell lines, including wild-type (WT) cells, or cells carrying knock-in mutation in FANCM helicase domain (D203A), or cells carrying deletion of its C-terminal domain that interacts with FAAP24, were treated with MMC (60 ng ml^−1^) for 18 h before being collected and analysis of FANCM focus formation. The graph in **c** shows the mean and standard deviations (s.d.) of the percentage of cells containing ⩾5 FANCM foci. The *P*-values between different cell lines are shown. MMC, mitomycin C.

When cells are treated with DNA-damaging drugs or under replication stress, many DNA repair proteins, including BLM and FANCD2, are re-distributed to the DNA damage sites or stalled replication forks, where they can be detected as large bright foci in the nuclei [12–14, 45]. However, it has been difficult to detect foci of FANCM in human cells following the treatment with DNA-reactive compounds, although we have been able to visualize recruitment of FANCM to laser-directed psoralen ICLs, because of the highly localized concentration of ICLs in the laser stripes [23]. To detect FANCM foci under regular drug-treated conditions, we generated an antibody against chicken FANCM, and found that this antibody readily detected FANCM in the bright nuclear foci in chicken DT40 cells treated with the drugs that induce replication stress, but not in the untreated cells (Figure 1a and b), or in *FANCM*^−/−^ cells treated with the same drugs (see Figure 2b below). These drugs include: mitomycin C (MMC), which induces ICLs that directly block replication forks; aphidicolin (APH), which inhibits DNA polymerase activity; and hydroxyurea, which depletes cellular nucleotide pools. The fact that FANCM-containing foci are induced by the drugs that cause different types of replication stress suggests that they represent recruitment of FANCM at stalled replication forks.

To further test this hypothesis, we investigated whether the FANCM foci co-localized with those of BLM and FANCD2 at stalled replication forks. Because of lack of appropriate antibodies against chicken proteins, we performed the analyses in *BLM*^*−/−*^ DT40 cells stably expressing green fluorescence protein (GFP)-tagged BLM (Supplementary Figure S1A and B) and in *FANCD2*^*−/−*^ DT40 cells stably expressing GFP-tagged FANCD2, respectively. We found that when these cells were treated with MMC, about 80% of FANCM and BLM foci (Figure 1c and d), and nearly 90% of FANCM and FANCD2 foci, co-localized with each other (Figure 1e and f), indicating that the recruitment of FANCM to stalled replication forks is similar to those of BLM and FANCD2.

### FANCM recruitment to stalled forks depends on its DNA translocase activity and its interaction with FAAP24

FANCM is an ATP-dependent DNA translocase that can remodel branched DNA, and this activity is critical for ATR activation, replication traverse of ICLs and SCE suppression [30, 32, 33, 36, 40, 41]. We therefore investigated if this activity is also needed for FANCM recruitment to stalled forks, by utilizing a DT40 cell line carrying a knock-in point mutation within the Walker B box of the FANCM helicase domain, FANCM-D203A (Figure 2a) [36]. We found that the percentage of cells containing FANCM foci was reduced in these cells (from about 50 to 8%; Figure 2b and c), suggesting that FANCM strongly depends on its translocase activity to be efficiently recruited to the sites of stalled forks. It should be pointed out that in the absence of its translocase activity, FANCM can still be recruited to stalled forks, albeit in a smaller percentage of cells (about 8%), suggesting that there are other mechanisms that recruit FANCM to stalled forks.

Next, we investigated whether FANCM recruitment depends on its two DNA-binding partners, FAAP24 and MHF (Figure 2a), both of which have been shown to stimulate *in vitro* and *in vivo* function of FANCM [22, 23]. For FAAP24, we utilized a *FANCM*^*−/−*^ DT40 cell line expressing a FANCM mutant lacking the C-terminal ERCC4-like nuclease domain FANCM-ΔC [36] (Figure 2a). This domain has been shown to directly interact with FAAP24 to form a heterodimer that has ssDNA binding activity but no nuclease activity, and deletion of the domain abolishes FANCM-FAAP24 association [22]. We found that these FANCM mutant cells (FANCM-ΔC) formed FANCM foci in response to MMC, but the percentage of cells with the foci was lower than that of wild-type cells (Figure 2b and c), suggesting that FANCM recruitment depends on its interaction with FAAP24. However, we cannot rule out the possibility that the reduced FANCM recruitment may be due to altered protein conformation in the FANCM-ΔC mutant, or due to loss of an unknown interacting partner that binds to the same region as FAAP24 does.

MHF binds to a motif adjacent to the helicase domain of FANCM (Figure 2a); and one of its subunits, MHF1, has been inactivated in DT40 cells [23]. We found that the percentage of *MHF1*^*−/−*^ cells that formed FANCM foci in response to MMC was lower when compared with that of the wild-type cells (about 8% vs 60%; Supplementary Figure S2A and B), suggesting that MHF may be needed for optimal recruitment of FANCM to forks stalled by ICLs. However, MHF is known to have at least two different effects on FANCM: it stabilizes FANCM protein, and provides a DNA-binding surface to help FANCM to bind DNA [23, 44]. To distinguish these possibilities, we rescued *MHF1*^*−/−*^ cells with an MHF1 point mutant A that is defective in DNA-binding but can stabilize FANCM [23]. We found that this mutant largely rescued FANCM recruitment to stalled forks when compared with the MHF1-wild-type protein (Supplementary Figure S2A–C), indicating that the observed reduction of FANCM foci is due to reduced FANCM stability in *MHF1*^*−/−*^ cells; and that the DNA-binding activity of MHF1 is dispensable for recruitment of FANCM to stalled forks.

### FANCM recruitment to stalled forks requires its association with BLM complex and BLM helicase activity

**Figure 3:**
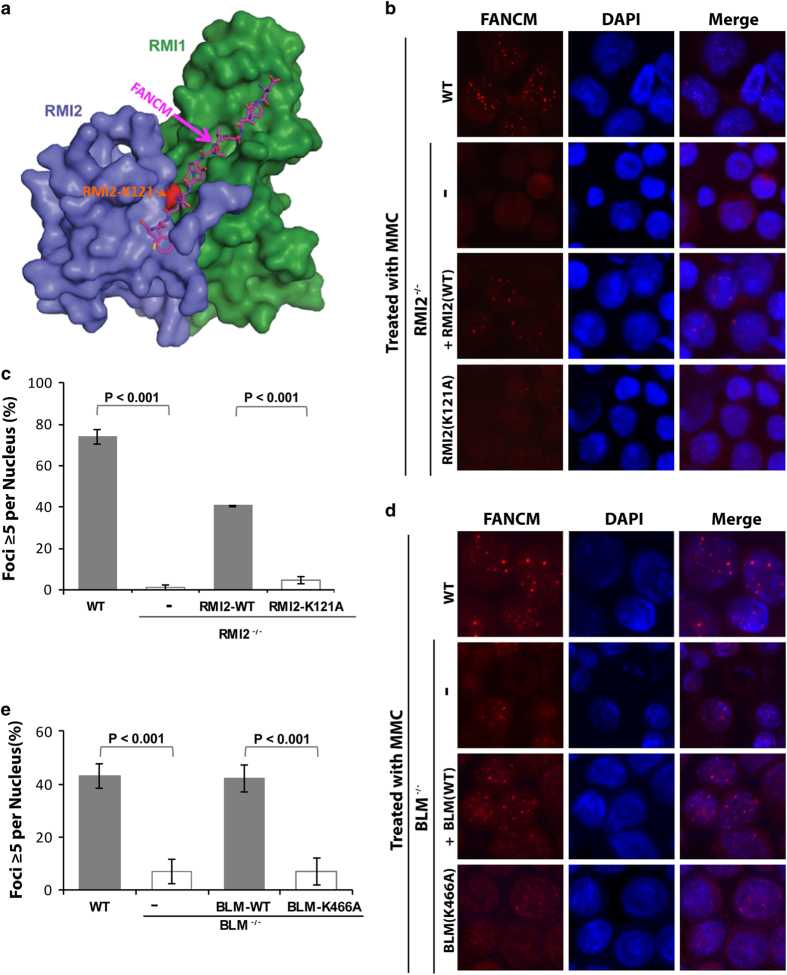
FANCM recruitment to stalled forks requires the RMI2-mediated association with the BLM complex and BLM helicase activity. (**a**) The overall structure of the RMI core complex with bound FANCM-MM2 peptide. The structure has been published in the previous publication [43], and is shown here for readers’ convenience. MM2 residues 1226–1237 are shown in pink. RMI1 is shown in green, RMI2 in blue and RMI2-K121 residue at the interface is highlighted in red [43]. (**b**) Immunofluorescence images and (**c**) quantification showing that MMC-induced focus formation of FANCM is defective in *RMI2* mutant DT40 cells. Various DT40 cell lines, including wild-type (WT) cells, *RMI2*^*−/−*^ cells, and *RMI2*^*−/−*^ cells complemented with either RMI2 wild-type (WT) or RMI2-K121A mutant, were treated with MMC (60 ng ml^−1^) for 18 h before harvest and analysis of FANCM foci. The graph in **c** shows the mean and standard deviations (s.d.) of the percentage of cells containing ⩾5 FANCM foci. The *P*-values between different cell lines are shown. (**d**, **e**) As described in **b**, **c**, respectively; except *BLM*^*−/−*^ cells and *BLM*^*−/−*^ cells complemented with either BLM wild-type protein or BLM-K466A helicase mutant were used. MMC, mitomycin C.

Previous studies have shown that recruitment of BLM to stalled forks requires its interaction with FANCM [35]. The findings prompted us to investigate whether recruitment of FANCM to stalled forks reciprocally depends on its interaction with the BLM complex. FANCM interacts with BLM complex through an interface consisting of residues from the MM2 motif of FANCM, RMI1 and RMI2 (Figures 2a and 3a) [35, 43]. It was shown that a single point mutation within RMI2, K121A, disrupts this interface, leading to dissociation between FANCM and BLM complex [43]. We found that the percentage of *RMI2*^*−/−*^ DT40 cells that form FANCM foci in response to MMC was drastically reduced when compared with that of the wild-type DT40 cells (Figure 3b and c); this reduction was largely rescued when human wild-type RMI2 was ectopically introduced into these cells (Figure 3b and c), indicating that RMI2 has a major role in recruiting FANCM to stalled forks. Notably, the introduction of RMI2-K121A point mutant into the same cells did not significantly rescue the reduced FANCM foci formation (Figure 3b and c), indicating that the RMI2-mediated association between FANCM and BLM complex is critical for recruitment of FANCM to stalled forks.

Next, we investigated whether BLM and its helicase activity are important for FANCM recruitment to stalled forks. We found that the percentage of *BLM*^*−/−*^ DT40 cells that formed FANCM foci in response to MMC was strongly reduced as compared with that of the wild-type cells (Figure 3d and e); and this reduction was recovered when GFP-tagged wild-type BLM protein was ectopically expressed in *BLM*^*−/−*^ cells, indicating that BLM has an important role in targeting FANCM to stalled forks. Notably, re-introduction of GFP-tagged BLM helicase mutant, K466A, failed to restore the reduction of FANCM foci formation (Figure 3d and e). The data suggest that the helicase activity of BLM is needed for normal FANCM recruitment to stalled forks. It should be noted that FANCM recruitment was reduced but not completely eliminated in *BLM*^*−/−*^ cells or in *BLM*^*−/−*^ cells expressing its helicase mutant, because about 5% of these cells contain more than five FANCM foci. The data suggest that FANCM recruitment to stalled forks can occur in the absence of BLM helicase activity, albeit at a lower efficiency.

### FANCM recruitment to stalled forks is independent of the FA core and FA ID complexes

**Figure 4:**
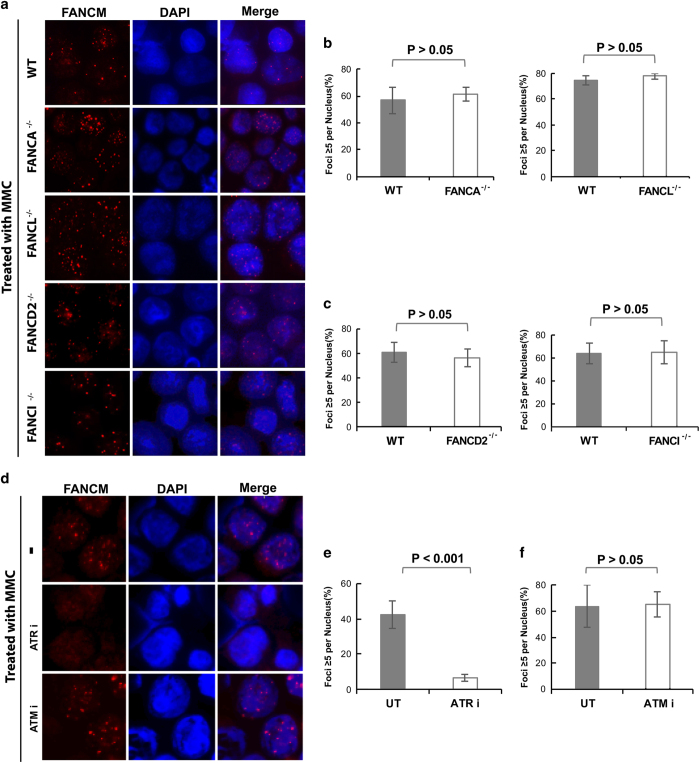
FANCM recruitment to stalled forks is independent of the FA core complex, FANCD2–FANCI complex and ATM, but is dependent on ATR. (**a**) Immunofluorescence images and (**b**, **c**) their quantification show that MMC-induced focus formation of FANCM is largely normal in DT40 cells carrying mutations in FANCA, FANCL, FANCD2 and FANCI. Various DT40 cell lines, including wild-type (WT), *FANCA*^*−/−*^, *FANCL*^*−/−*^, *FANCD2*^*−/−*^ and *FANCI*^*−/−*^ cells, were treated with MMC (60 ng ml^−1^) for 18 h before harvest and analysis of FANCM foci. The graphs in **b**, **c** show the mean and standard deviations (s.d.) of the percentage of cells containing ⩾5 FANCM foci. The *P*-values between different cell lines are shown on the top. (**d**) Immunofluorescence images and (**e**, **f**) their quantification show that focus formation of FANCM was reduced in DT40 cells pretreated for 2 h with an ATR inhibitor (VE821 at 0.6 μm), but not with an ATM inhibitor (KU55933 at 10 μm). After pretreatment, the concentration of inhibitors was reduced by half and the cells were treated with MMC (50 ng ml^−1^) for 18 h. The cells without treatment were used as a control. FA, Fanconi anemia; MMC, mitomycin C.

FANCM directly interacts with the FA core complex (Figure 2a), and it is also required for recruitment of the FA core complex to chromatin and stalled forks [19, 35, 46, 47]. We investigated whether the FA core complex is reciprocally needed for FANCM recruitment to stalled forks. It was shown that the subunit of the FA core complex that directly interacts with FANCM is FANCF [35]. However, FANCF-knockout DT40 cell line is currently not available. Thus, we chose to examine FANCM recruitment in DT40 cells inactivated of two FA core complex subunits, FANCA and FANCL, because both have been shown to be crucial for stability and assembly of the FA core complex [19, 21, 48, 49]. FANCA inactivation destabilizes FANCG and also impairs nuclear localization of FANCL, FANCB and FAAP100; whereas FANCL inactivation destabilizes FAAP100, and also impairs the association among FANCF, FANCA and FANCG [19, 21, 48, 49]. We found that the percentage of *FANCA*^*−/−*^ and *FANCL*^*−/−*^ cells that form FANCM foci in response to MMC was indistinguishable from that of the wild-type cells (Figure 4a and b), suggesting that FANCM recruitment to stalled forks is independent of the FA core complex.

We also examined whether FANCM recruitment to stalled forks depends on two FA proteins working downstream of the FA pathway, FANCD2 and FANCI. We found that for DT40 cells inactivated of either FANCD2 or FANCI, the percentage of cells that formed FANCM foci in response to MMC was comparable to that of wild-type cells (Figure 4a and c), indicating that FANCM recruitment to stalled forks is independent of the FA ID complex.

### FANCM recruitment to stalled forks depends on ATR but not ATM

FANCM is hyperphosphorylated in response to DNA damage and replication stress; and this phosphorylation has been reported to depend on cell cycle checkpoint kinases, ATR and ATM [19, 50, 51]. We found that, when DT40 cells were treated with an ATR kinase inhibitor, VE821, the percentage of cells that form FANCM foci in response to MMC was decreased by about 5-fold (Figure 4d and e), suggesting that FANCM recruitment to stalled forks depends on ATR-mediated phosphorylation. In contrast, when the same cells were treated with an ATM kinase inhibitor, KU55933, the percentage of FANCM foci-positive cells was comparable to that of wild-type cells (Figure 4d and f). The data suggest that recruitment of FANCM to stalled forks depends on ATR, but not on ATM, which is parallel to the earlier results that ATR-dependent phosphorylation of FANCM is required for its recruitment to sites of ICLs regardless of cell cycle stages [51].

As a control experiment, we investigated whether ATR, or ATM, or both, were activated by replication stress in DT40 cells. We found that when DT40 cells were treated with drugs that induce replication stress, such as MMC or aphidicolin, a major downstream target of ATR, chk1, became robustly hyperphosphorylated (Supplementary Figure S3). Moreover, the MMC-induced chk1 hyperphosphorylation was strongly reduced when the ATR inhibitor was used to treat these cells (Supplementary Figure S4A and B, lanes 1, 3 and 5). In contrast, a major downstream target of ATM, chk2, did not show obvious hyperphophorylation by either MMC or aphidicolin treatment; and addition of the ATM inhibitor had no obvious effect on chk2 or chk1 hyperphosphorylation (Supplementary Figure S3, compare lanes 5 and 6 to 2 and 3). The results suggest that only ATR, but not ATM, was significantly activated by replication stress under our conditions. The data are consistent with the earlier findings that ATR is mainly activated by replication stress, whereas ATM by double-strand breaks [52].

### FANCM hyperphosphorylation in response to replication stress requires its association with BLM complex

**Figure 5:**
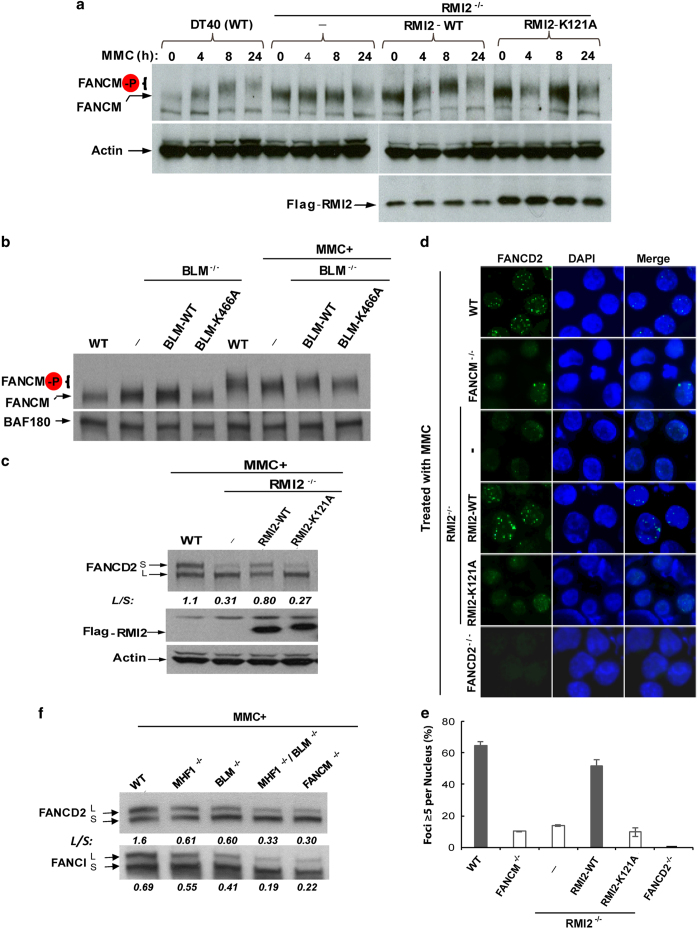
RMI2-mediated association between FANCM and BLM complex is required for FANCM hyperphosphorylation, FANCD2 monoubiquitination and foci formation. (**a**) Immunoblotting shows that FANCM hyperphosphorylation and FANCD2 monoubiquitination are concomitantly reduced in MMC-treated *RMI2*^*−/−*^ cells, or *RMI2*^*−/−*^ cells expressing RMI2-121A point mutant. Wild-type, or *RMI2*^*−/−*^ cells, or *RMI2*^*−/−*^ cells complemented by either wild-type RMI2 or 121A point mutant [43], were treated with MMC (50 ng ml^−1^) for increasing lengths of time, as indicated above the images. They were then collected for western analyses. The monoubiquitinated and non-ubiquitinated FANCD2 was indicated as FANCD2-L and S (long and short), whereas hyperphosphorylated FANCM was marked as FANCM-P and was blotted from 6% gel with extended electrophoresis. The mobility of FANCM is decreased in response to MMC due to hyperphosphorylation [19]. (**b**) Immunoblotting shows that the level of hyperphosphorylated FANCM is modestly reduced in BLM mutant DT40 cells treated with MMC (50 ng ml^−1^) for 18 h. Immunoblotting of BAF180 (a subunit of PBAF chromatin remodeling complex) was included as a loading control. (**c**) Immunoblotting shows the level of monoubiquitinated FANCD2 is reduced in *RMI2*^*−/−*^ cells, or in the same cells expressing RMI2-K121A mutant. The cells were all treated with MMC (50 ng ml^−1^) for 18 h. The ratio between the monoubiquitinated and ubiquitinated FANCD2 (L/S) was shown. (**d**) Representative images and (**e**) their quantification show that MMC-induced FANCD2 focus formation is reduced in MMC-treated *RMI2*^*−/−*^ cells, or *RMI2*^*−/−*^ cells expressing RMI2-121A point mutant. Various DT40 cell lines were treated with MMC (60 ng ml^−1^) for 18 h before they were collected for the analyses of FANCD2 foci. The cells include wild-type (WT), *RMI2*^*−/−*^, *RMI2*^*−/−*^ cells complemented with RMI2 wild-type (WT) or RMI2-K121A mutant, *FANCM*^*−/−*^ and *FANCD2*^*−/−*^ cells. The latter two cells were included as controls. The graph in **e** shows the mean and standard deviations (s.d.) of the percentage of cells containing ⩾5 FANCD2 foci, as assayed in **d**. The *P*-values between different cell lines are shown. (**f**) Immunoblotting shows the levels of monoubiquitinated FANCD2 and FANCI in various DT40 cell lines as indicated. The cells were all treated with MMC (50 ng ml^−1^) for 18 h. The ratios between the monoubiquitinated and ubiquitinated FANCD2 or FANCI (L/S) were shown. MMC, mitomycin C.

The findings above that FANCM recruitment to stalled forks require both its association with the BLM complex and ATR-dependent phosphorylation raised a possibility that the two processes may be linked. We therefore investigated whether FANCM hyperphosphorylation in response to replication stress requires its association with BLM complex, using SDS polyacrylamide gel electrophoresis that can distinguish the hyperphosphorylated from hypophosphorylated forms of FANCM [19, 51]. Consistent with earlier findings, FANCM in wild-type DT40 cells treated with MMC for increasing lengths of time exhibited decreasing mobility on SDS gels (Figure 5a), indicating that more FANCM became hyperphosphorylated when more DT40 cells entered S-phase where the cells were subject to MMC-induced replication stress. In contrast, FANCM from *RMI2*^*−/−*^ cells treated with MMC failed to exhibit noticeable mobility decrease; and this failure was corrected when human wild-type RMI2 was re-introduced into these cells (Figure 5a). The data indicate that replication stress-induced FANCM hyperphosphorylation requires RMI2. Notably, the decrease of FANCM mobility was not restored by re-introduction of the RMI2-K121A mutant, which disrupts FANCM–BLM complex association (Figure 5a) [43]. These results demonstrate that replication stress-induced FANCM hypersphosphorylation depends on RMI2-mediated interaction between FANCM and BLM complex.

As a comparison, we examined FANCM hyperphosphorylation in *BLM*^*−/−*^ DT40 cells treated with MMC, and observed apparent decrease in FANCM gel mobility when compared to that of untreated cells, suggesting that FANCM hyperphosphorylation occurs in the absence of BLM. However, the extent of this decrease was slightly smaller than that of wild-type cells, or *BLM*^*−/−*^ cells complemented by exogenously introduced wild-type BLM (Figure 5b), suggesting that FANCM hyperphosphorylation was modestly reduced by the absence of BLM. We also examined FANCM mobility in *BLM*^*−/−*^ cells complemented by BLM helicase mutant, K466A, and found it was similar to that of *BLM*^*−/−*^ cells (Figure 5b), suggesting that optimal FANCM hyperphosphorylation in response to MMC depends on BLM helicase activity.

ATR is the major replication checkpoint kinase and phosphorylates many substrates when cells are under replication stress. The finding that ATR-mediated phosphorylation of FANCM depends on the BLM complex prompted us to investigate whether ATR-mediated phosphorylation of chk1 has the same dependence. We observed robust MMC-induced chk1 hyperphosphorylation in both *BLM*^*−/−*^ and *RMI2*^*−/−*^ DT40 cells when compared with their untreated cells (Supplementary Figure S4A and B; lanes 4 vs 2); and the levels of the hyperphosphorylated chk1 were comparable to those of the wild-type cells (Supplementary Figure S4A and B, lanes 3 and 4). Notably, addition of the ATR inhibitor reduced the hyperphosphorylated levels of chk1 (Supplementary Figure S4A and B, lane 6 vs 4). These data suggest that ATR-mediated phosphorylation of chk1 (and possibly other substrates) does not depend on the BLM complex.

### The chromatin association of FANCM does not depend on the BLM complex or its phosphorylation statues

FANCM is known to exclusively associate with chromatin, and this association depends on FAAP24 and MHF [23, 47]. Our findings that BLM complex is needed for MMC-induced FANCM hyperphosphorylation and recruitment to stalled replication forks led us to investigate whether the BLM complex is also required for chromatin association of FANCM. Consistent with previous results, we found that FANCM was largely present in the chromatin fractions in MMC-treated wild-type DT40 cells (Supplementary Figure S5A and B, lanes 5 vs 1). Notably, in both *RMI2*^*−/−*^ and *BLM*^*−/−*^ cells, and in these mutant cells rescued by re-introduction of the corresponding wild-type and mutant proteins, most of FANCM was always present in the chromatin fractions (Supplementary Figure S5A and B, lanes 6–8 vs 2–4), indicating that the BLM complex is dispensable for FANCM chromatin association.

We noticed that FANCM in chromatin isolated from the MMC-treated wild-type cells, or the mutant cells rescued by the wild-type protein, is mainly in hyperphosphorylated form (Supplementary Figure S5A and B, lanes 5 and 7). In contrast, FANCM in chromatin isolated from MMC-treated *RMI2*^*−/−*^ cells, *RMI2*^*−/−*^ cells rescued by the K121A mutant, *BLM*^*−/−*^ cells and *BLM*^*−/−*^ cells rescued by its helicase mutant, is mainly in hypophosphorylated form (Supplementary Figure S5A and B, lanes 6 and 8). The data support our findings that BLM complex is needed for FANCM hyperphosphorylation in response to replication stress (Figure 5). Moreover, because the amount of hyper- and hypo- phosphorylated FANCM in chromatin was comparable, the data suggest that the phosphorylation statues of FANCM does not significantly alter its chromatin association.

### RMI2-mediated FANCM–BLM association promotes activation of the FA pathway

Mutation in FANCM phosphorylation sites has been shown to disrupt FANCD2 monoubiquitination and foci formation in response to replication stress [51], both of which are key steps of the FA pathway. Because RMI2 mutant cells lacking FANCM–BLM association are defective in FANCM hyperphophorylation, we hypothesize that the same cells may also be impaired in the FA pathway. Consistent with this hypothesis, both the monoubiquitinated FANCD2 level (Figure 5c) and the percentage of cells with FANCD2 foci (Figure 5d and e) were reduced in *RMI2*^*−/−*^ DT40 cells treated with MMC and these reductions were largely rescued by re-expression of wild-type RMI2 in the same cells (Figure 5c–e). These data suggest that RMI2 is needed for normal activation of the FA pathway. Notably, the reduced FANCD2 monoubiquitination and foci formation were not rescued when RMI2-K121A mutant was re-expressed in the same cells (Figure 5c–e), indicating that RMI2-mediated FANCM–BLM association is necessary for normal activation of the FA pathway.

Previous studies have reported that FANCD2 monoubiquitination was modestly reduced in BLM-deficient human cells [53] and *BLM*^*−/−*^ chicken DT40 cells in response to drugs that induce ICLs [36]. We performed the experiments in *BLM*^*−/−*^ DT40 cells and observed a similar reduction (Figure 5f and Supplementary Figure S1A). Interestingly, we found that in DT40 cells inactivated of both BLM and MHF1, the level of monoubiquitinated FANCD2 and FANCI was further reduced when compared with that of each single mutant cell line; and this level was comparable to that of *FANCM*^*−/−*^ cells (Figure 5f and Supplementary Figure S1C and D). Because both BLM complex and MHF interact with FANCM through different motifs, our data imply that they may work in parallel pathways to help FANCM in activation of the FA pathway.

### RMI2-mediated FANCM–BLM association is required for replication traverse of ICLs

**Figure 6:**
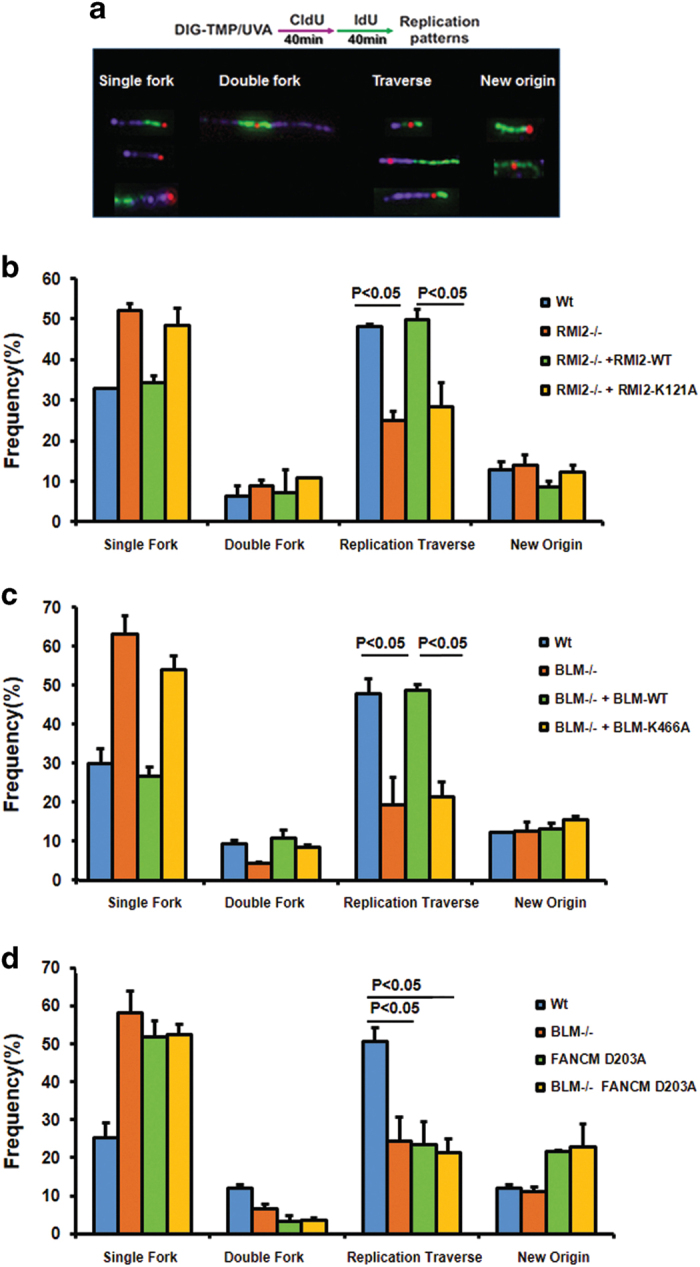
BLM complex and FANCM work in the same pathway to promote replication traverse of ICLs. (**a**) Patterns of replication tracts in the vicinity of Dig-TMP ICLs (red dot) on DNA fibers. The protocol of this assay is illustrated on the top. The sequence of the differentially colored CldU (purple) and IdU (green) tracks defines the direction of replication forks. (**b**) Frequency of patterns in wild-type DT40 cells, RMI2 knockout DT40 cells and RMI2 knockout cells complemented with either the wild-type *RMI2* gene or the RMI2 with K121A point mutant. (*n*=61, 70, 64 in the wild-type cells; 51, 75, 69 in RMI2-deficient DT40 cells; 63, 54, 52 in RMI2-deficient cells complemented with either the wild-type; 46, 74, 60 in the RMI2 K121A point mutant cells. ‘*n*’ indicates the number of encounters analyzed in individual experiments) (**c**) Frequency of patterns in wild-type DT40 cells, BLM-deficient DT40 cells and BLM-deficient cells complemented with either the wild-type *BLM* gene or the BLM with K466A mutant. Three independent experiments were performed. (*n*=75, 83, 62 in the wild-type cells; 45, 63, 74 in BLM-deficient DT40 cells; 63, 64, 52 in BLM-deficient cells complemented with either the wild-type BLM; 66, 74, 51 in the BLM K466A point mutant cells). (**d**) Frequency of patterns in wild-type DT40 cells, BLM-deficient DT40 cells, FANCM-deficient cells expressing FANCM D203A mutant and BLM knockout DT40 cells expressing FANCM D203A mutant (*n*=61, 78, 84 in the wild-type cells; 81, 75, 68 in BLM-deficient DT40 cells; 73, 64, 62 in FANCM-D203A mutant cells; 66, 84, 70 in the BLM^−/−^ /FANCM-D203A cells). Dig-TMP, digoxigenin-tagged trimethylpsoralen; ICL, interstrand crosslink; MMC, mitomycin C.

FANCM has a major role in promoting replication traverse of ICLs [30]. Because RMI2-mediated FANCM–BLM association is required for FANCM hyperphosphorylation and recruitment to stalled forks, we studied whether this association is needed for replication traverse of ICLs using the same assay described previously [30]. Briefly, DT40 cells of different genotypes were first treated with Dig-TMP (digoxigenin-tagged trimethylpsoralen) and ultraviolet A to induce ICLs; and then were sequentially pulsed with CIdU and IdU to label replication tracks (Figure 6a). *RMI2*^*−/−*^ DT40 cells showed a lower level of replication traverse compared with that of the wild-type cells (about 30% vs 50%); and re-expression of the wild-type RMI2 protein restored the traverse level to that of wild-type cells (about 50%; Figure 6b), indicating that RMI2 is important for replication traverse of ICLs. Notably, re-expression of the RMI2-K121A mutant failed to restore the traverse level to that of wild-type cells (it remained at about 30%), indicating that RMI2-mediated FANCM–BLM association is important for the replication machinery to traverse the ICLs.

### Both BLM and its helicase activity are required for replication traverse of ICLs

We next investigated whether BLM and its helicase activity are required for replication traverse using the same assay as above. We observed that *BLM*^*−/−*^ DT40 cells displayed a reduced level of traverse than wild-type type cells (about 20% vs 50%); and re-introduction of wild-type BLM in these cells restored the level to that of the wild-type cells (Figure 6c), suggesting that BLM is needed for normal traverse of ICLs. Re-introduction of BLM helicase mutant, K466A, failed to restore the traverse level to that of wild-type cells (it remained at about 20%), indicating that BLM requires its helicase activity to promote replication traverse of ICLs.

### BLM complex and FANCM work in the same pathway to promote replication traverse of ICLs

Both BLM and FANCM can remodel branched DNA structures, including forks, using their helicase and translocase activity, respectively. Earlier studies have shown that the two proteins work in the same pathway to suppress SCEs and to promote cellular resistance to ICLs [36]. We studied how they act in the replication traverse pathway. Consistent with earlier findings [30], DT40 cells carrying a knock-in mutation of the FANCM helicase domain, D203A, displayed a lower level of traverse than wild-type DT40 cells (about 20% vs 50%); and this level was comparable to that of *BLM*^*−/−*^ cells (Figure 6d). Notably, the double-mutant cells showed a level of traverse indistinguishable from that of each single mutant (Figure 6d), indicating that the two proteins work in the same pathway to promote replication traverse.

BLM and FANCM have been shown to suppress new origin firing in human and/or chicken DT40 cells [15, 30, 54]. We obtained similar findings in our analyses for FANCM-D203A mutant cells (Figure 6d), but we did not observe an obvious increase of new origin firing in *BLM*^*−/−*^ cells. One possible explanation for this difference is that we used a DNA crosslinking drug to induce replication stress, whereas the prior studies used non-crosslinking drugs [15]. BLM may only be needed for suppressing new origin firing for the latter drugs.

## Discussion

### The recruitment of FANCM depends on its translocase activity, DNA-binding partners and phosphorylation by ATR

**Figure 7:**
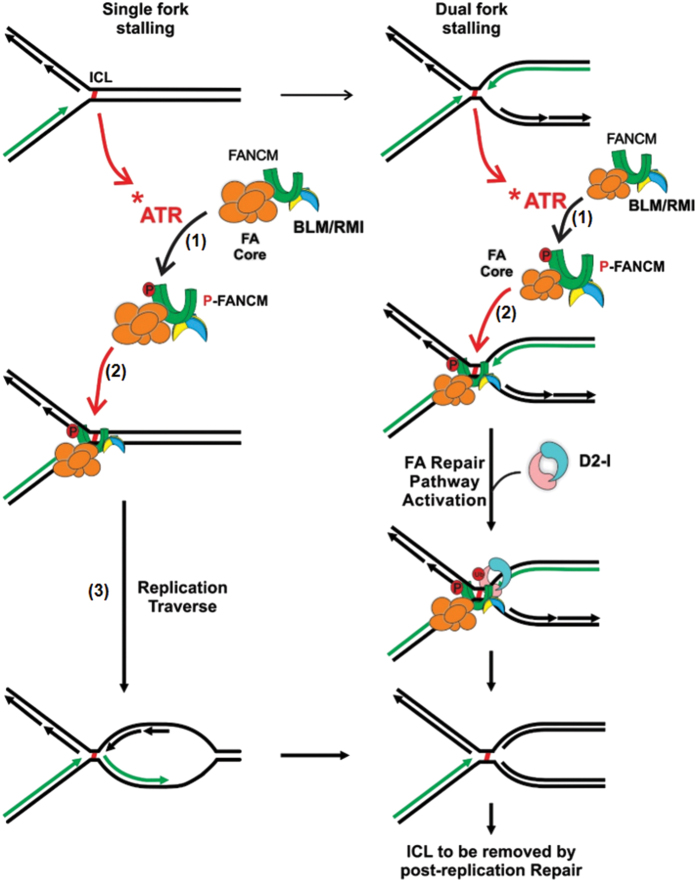
A model on how BLM complex promotes FANCM recruitment to stalled forks and replication traverse of ICLs. A cartoon illustrates that the recruitment of FANCM to stalled forks is an event downstream of ATR but upstream of both FA repair pathway or the replication traverse pathway to repair or traverse ICLs. Two different scenarios when replication forks collide with ICLs (single fork-stalling and double-folk stalling) [30] are shown. The stalled single forks can continue past ICLs in the FANCM-dependent traverse pathway [30], whereas the stalled double-forks can initiate Fanconi anemia pathway to repair the ICLs [73]. BLM complex can act at multiple steps in the two pathways. It may enhance FANCM hyperphosphorylation (step 1) by direct protein–protein interactions. Alternatively, it may promote FANCM recruitment to stalled forks using its DNA binding and helicase activity (step 2). Moreover, it may stimulate the downstream traverse reaction using BLM helicase activity (step 3). FANCM is required for recruitment of FA core complex (marked by FANCA) and BLM to stalled forks [35]. Because FANCM, BLM and FA core complex form a highly stable multi-subunit complex [7, 18, 19, 23, 35], they may be co-recruited to stalled forks. Their recruitment depends on DNA translocase activity of FANCM and helicase activity of BLM, which may explain the observation that FANCM and BLM mutually depend on each other for their recruitment [35] (this study), and FA core complex depends on FANCM for its recruitment [46]. ‘D2-I’ refers to the FANCD2–FANCI complex, which is monoubiquitinated by the FA core complex in response to replication stress. The ubiquitination is marked by ‘Ub’, hyperphosphorylation is marked by ‘P’. FA, Fanconi anemia; ICL, interstrand crosslink; MMC, mitomycin C.

In this study, we elucidated the mechanism of a key step in ICL-induced DNA damage response pathways—the FANCM recruitment to stalled replication forks. We demonstrate that this recruitment not only needs its intrinsic activity, but also strongly depends on its direct interaction with external factors, such as BLM complex. First, the FANCM recruitment depends on its own translocase activity, which is necessary for replication traverse of ICLs [30]. Thus, targeting FANCM to stalled forks could be a new mechanism by which FANCM translocase facilitates the traverse of ICLs. Possibly, translocating FANCM on dsDNA may enable FANCM to scan large regions of genomes and locate the stalled forks. Second, the FANCM recruitment depends on ATR, which is known to hyperphosphorylate FANCM [51] in response to replication stress. This feature of FANCM resembles that of FA core complex [55], FANCD2 [56, 57] and BLM [58], the recruitment of which also depends on ATR. Our data thus suggest that FANCM recruitment occurs downstream of ATR, which is similar to that of the FA core complex [46] (Figure 7).

Several independent studies have shown that FANCM and its partner, FAAP24, are required for full ATR activation in response to replication stress [31, 34, 40, 59]. Conversely, other studies, including this one, have shown that FANCM is a downstream substrate of ATR [50, 51]. Together, these data imply that ATR and FANCM may constitute a positive feedback loop that mutually activates each other. This loop may not only sense and transduce the stress signal, but also amplifies it, to elicit a stronger response downstream. One question is which one is activated first? We hypothesize that ATR is likely to be activated first, based on the fact that ATR is essential for viability of mouse and many cell lines [60], whereas FANCM is non-essential [61]. Thus, there may exist FANCM-independent pathways that can activate ATR.

Because the FA core complex and FANCM can be co-purified in a highly stable complex [7, 19, 23], they are likely to be co-recruited to stalled forks. However, the association notwithstanding, FANCM recruitment does not depend on the core complex, suggesting that the core complex is a passive partner during FANCM recruitment, after which it monoubiquitinates FANCD2. Notably, the FANCM recruitment depends on its DNA-binding partners, FAAP24 and BLM complex; but not on the FA core complex, which lacks obvious DNA-binding activity [7]. FAAP24 is known to stimulate FANCM to bind DNA *in vitro,* and is required for FANCM to localize to chromatin and damaged DNA *in vivo *[22, 47], so that its contribution to FANCM recruitment was to be expected. However, the roles of BLM complex in FANCM recruitment and function have not been addressed before, and will be discussed below.

### BLM complex and FANCM are coordinately recruited to stalled forks

We have detected the recruitment of FANCM to stalled replication forks where it co-localizes with BLM and FANCD2. Because BLM can be purified as a stable complex with FANCM and FA core complex [7, 23], and FANCM can simultaneously interact with both BLM complex and FA core complex using two separate motifs [35, 62], our findings imply that BLM is co-recruited with FANCM and FA core complex to stalled forks as a super-complex (Figure 7). Consistent with this notion, earlier studies have shown that BLM recruitment requires its association with FANCM [35]. Moreover, FA core complex recruitment depends on FANCM [46]. Furthermore, our data showed that FANCM recruitment reciprocally requires its association with BLM complex. This mutual dependence supports their co-recruitment: when a DNA-binding component of the super-complex (BLM or FANCM) is absent, the recruitment of other components is defective.

How may BLM complex stimulate FANCM recruitment to stalled forks? Our data suggest that this stimulation can occur by at least two possible mechanisms. One, BLM complex may stimulate FANCM hyperphosphorylation through RMI2-mediated protein–protein interactions. Two, BLM may also enhance FANCM recruitment using its helicase activity. The evidence includes that FANCM recruitment is reduced either by RMI2-K121A mutation, which disrupts BLM–FANCM association; or by BLM helicase mutation. Notably, FANCM hyperphosphorylation appears to be more impaired by the former than the latter mutation (Figure 5). One explanation for this difference is that the BLM complex contains two proteins that can bind DNA, BLM and Top3a. The RMI2-K121A mutation dissociates both from FANCM, whereas the BLM mutation dissociates only one, so that the former mutation should have a stronger effect than the latter on FANCM hyperphosphorylation. Consistent with this notion, RMI2-K121A mutant cells also have lower level of monoubiquitinated FANCD2 than *BLM*^*−/−*^ cells. The data suggest that multiple components of the BLM complex may contribute to FANCM hyperphosphorylation, recruitment and possibly other functions.

### BLM works with FANCM to promote both FA repair and replication traverse pathways

Among those of FANCM-interacting partners, MHF resembles FANCM in that both are required for optimal execution of the FA repair and replication traverse pathways for ICLs, whereas the FA core complex is needed only for the former but not for the latter (See Figure 7) [23, 24, 30]. BLM has been previously implicated in the FA pathway and repair of ICLs [36, 53, 63–65]. This study uncovered a new role of the BLM complex—it works with FANCM in the traverse pathway (Figure 6). Importantly, our data demonstrate that BLM complex may coordinate the two pathways at different steps using different mechanisms (Figure 7). First, the RMI2-mediated interactions between BLM complex and FANCM may trigger an earlier step upstream of the two pathways—FANCM hyperphosphorylation (Figure 7). Second, BLM may apply its helicase activity and DNA-binding activity to facilitate another upstream event—recruitment of FANCM to stalled forks. Top3a of the BLM complex may contribute to this step using its DNA-binding activity, in a manner similar to FAAP24, which increase binding of FANCM to DNA *in vitro* and to stalled forks *in vivo*. The increased FANCM recruitment may stimulate both FA repair and traverse pathways downstream. Third, BLM may utilize its helicase activity to specifically enhance the traverse pathway. In this regard, BLM may use a mechanism similar to that of FANCM, which uses its translocase activity to specifically promote the traverse pathway, but not FANCD2 monoubiquitination.

In summary, our studies revealed new roles for BLM complex in promoting FANCM recruitment to stalled replication forks, which leads to activation of both repair and bypass pathways for ICLs.

## Materials and Methods

### Cell lines

Chicken DT40 cells were cultured in RPMI-1640 medium supplemented with 10% fetal calf serum, 1% chicken serum, 2 mm l-glutamine and 10 mm HEPES in a 5% CO_2_ incubator at 39.5 °C. The chicken DT40 cell lines, including wild-type, *FANCM*^*−/−*^, *FANCM* knock-in mutants carrying C-terminal deletion or D203A point mutation [36]; *RMI2*^*−/−*^, *RMI2*^*−/−*^ complemented with RMI2 wild-type or carrying K121A mutation [10]; *FANCD2*^*−/−*^ [66], *FANCD2*^*−/−*^complemented with GFP-chicken FANCD2 [67]*; FANCL*^*−/−*^ [68], *FANCI*^*−/−*^ [69] and *MHF1*^*−/−*^ [23], have been previously described. The *FANCA*^*−/−*^ cells were generated by integrating a FANCA-targeting vector that can replace exons 7 and 8 with a *bsr*- or *his*-resistant gene cassette of the genomic fragment of chicken *FANCA* gene, which was isolated by PCR amplification from DT40 genomic DNA [70]. The *BLM*^*−/−*^ cells were generated by integrating a BLM-targeting vector into DT40 cells as previously described [71]. GFP-chicken BLM-wt [63] and K466A mutant expression vectors were introduced into *BLM*^*−/−*^ cells, and transfections and selection of the clones were done as published procedures [66].

### Antibodies and other reagents

An anti-chicken FANCM polyclonal rabbit antibody (amino acids 773–879) was generated and purified with the method as previous described [19]. An anti-chicken FANCD2 antibody was previously described [72]. An anti-Flag antibody (Sigma-Aldrich, St Louis, MO, USA), anti-GFP antibody (Sigma-Aldrich), anti-actin antibody (Bethyl Laboratory, Motgomery, TX, USA), anti-α-Tubulin antibody (Cell Signaling, Danvers, MA, USA), anti-Chk1 antibody (Santa Cruz Biotechnology, Santa Cruz, CA, USA), anti-phosphorylated Chk1 (phosphorylated S345) antibody (Cell Signalling), anti-Chk2 antibody (BD Biosciences, San Jose, CA, USA), anti-phosphorylated Chk2 (phosphorylated T68) antibody (Novus Biologicals, Littleton, CO, USA), anti-GAPDH (14C10, Cell Signalling), Histone H3 antibody (EMD Millipore, Billerica, MA, USA). ATR inhibitor VE821 (Selleck Chemicals, Houston, TX, USA), ATM inhibitor KU55933 (Abcam, Cambridge, MA, USA), MMC (Sigma-Aldrich), hydroxyurea (Sigma-Aldrich) and APH (Sigma-Aldrich) were purchased.

### Focus formation assay

DT40 cells of different genotypes were either untreated or treated with MMC (60 ng ml^−1^), APH (1.25 μg ml^−1^) or hydroxyurea (1.5 μm) for 18 h. They were then collected and washed with phosphate-buffered saline before cytospin. The cells were fixed with 4% paraformaldehyde for 10 min and permeabilized with 0.2% NP40 for 10 min. They were then either blocked for 1 h and probed for 1 h using a chicken FANCD2 antibody (1:1 000) in blocking buffer (5% goat serum, 0.2% Triton, 1× phosphate-buffered saline) at room temperature; or fixed with cold methanol for 5 min, and then, blocked with the same buffer for 1 h and probed for 2 h in chFANCM antibody (1:200) at room temperature. After washing with 0.05% tween phosphate-buffered saline buffer for three times, the cells were incubated with either anti-rabbit Alexa Fluor 488 Conjugate or 594 Conjugate antibodies for 1 h at room temperature. They were then washed and mounted with ProLong Gold Antifade Mountant (Invitrogen #P36934, Carlsbad, CA, USA). Images were taken with Zeiss 200 microscope, and nuclei with five or more bright foci were scored as positive. All the experiments were independently repeated at least two times. More than 200 nuclei were scored for each cell line.

### Cellular extraction, subcellular fractionation and immunoblotting

The cell lysis buffer (10 mm Tris-HCl at pH 7.5, 0.1% SDS, 0.1% sodium deoxycholate, 150 mm NaCl, 1% NP40, 1 mm EDTA and a complete protease inhibitor cocktail (Roche, Indianapolis, IN, USA)) was used for the preparation of whole-cell extract. The fractionation of cells into chromatin and soluble fractions has been described [23]. Briefly, a low-salt buffer (10 mm Tris-HCl pH 7.5, 10 mm NaCl, 3 mm MgCl_2_, 1 mm EDTA, 0.5% NP40, and a complete protease inhibitor cocktail (Roche)) was added to the cell pellets and incubated 5 min on ice. The cells were then centrifuged at 5K r.p.m. for 5 min to obtain the Soluble Fraction (the supernatant fraction). The pellet was extracted with a urea-containing buffer (8 m urea, 0.1 m NaH_2_PO_4_, 0.02 m Tris-HCl, pH 8.0). The pellet was spun down at 15K r.p.m. for 10 min. The supernatant was saved as the chromatin fraction. FANCM phosphorylation was detected with 6% Tris-glycine SDS gels (Invitrogen) or 4–15% Mini-PROTEIN TGX gels (Bio-Rad, Hercules, CA, USA). FANCD2 ubiquitination analyses were carried out with 6% Tris-glycine gels.

### Replication traverse assay.

The assay was done as previously reported [30]. Briefly, the cells were treated with 5 μm Dig-TMP for 1 h and exposed to ultraviolet A irradiation in a Rayonet (Brandford, CT, USA) chamber at 3 J cm^−2^. They were then incubated with 20 μm CldU for 40 min and then for 40 min with 100 μm IdU. The cells were lysed with 0.5% SDS in 200 mm Tris/HCl, 50 mm EDTA, pH 7.5 on a silanated glass slide (Newcomer Supply, Middleton, WI, USA). After tilting, the slides were air dried and fixed in 3:1 methanol/acetic acid, incubated in 2.5 m HCl for 60 min, neutralized in 0.4 m Tris/HCl, pH 7.5 for 5 min and immunostained with antibodies against Digoxigenin, CldU and IdU. Imaging was performed on a Zeiss (Oberkochen, Germany) Axiovert 200M microscope. Three independent experiments were performed. The number of encounters analyzed in individual experiments are shown in the figure legend.
