# Supplementary material for: Bloom syndrome complex promotes FANCM recruitment to stalled replication forks and facilitates both repair and traverse of DNA interstrand crosslinks
Source: Cell Discov. 2016 Dec 20;2:16047–. doi: 10.1038/celldisc.2016.47 (PMC5167996; doi:10.1038/celldisc.2016.47)
Supplement: Supplementary Information [file celldisc201647-s1.pdf]

Ling et al. Supplementary Figure 1.

Generation of knockout DT40 cell lines and GFP-BLM stably expressed cell lines

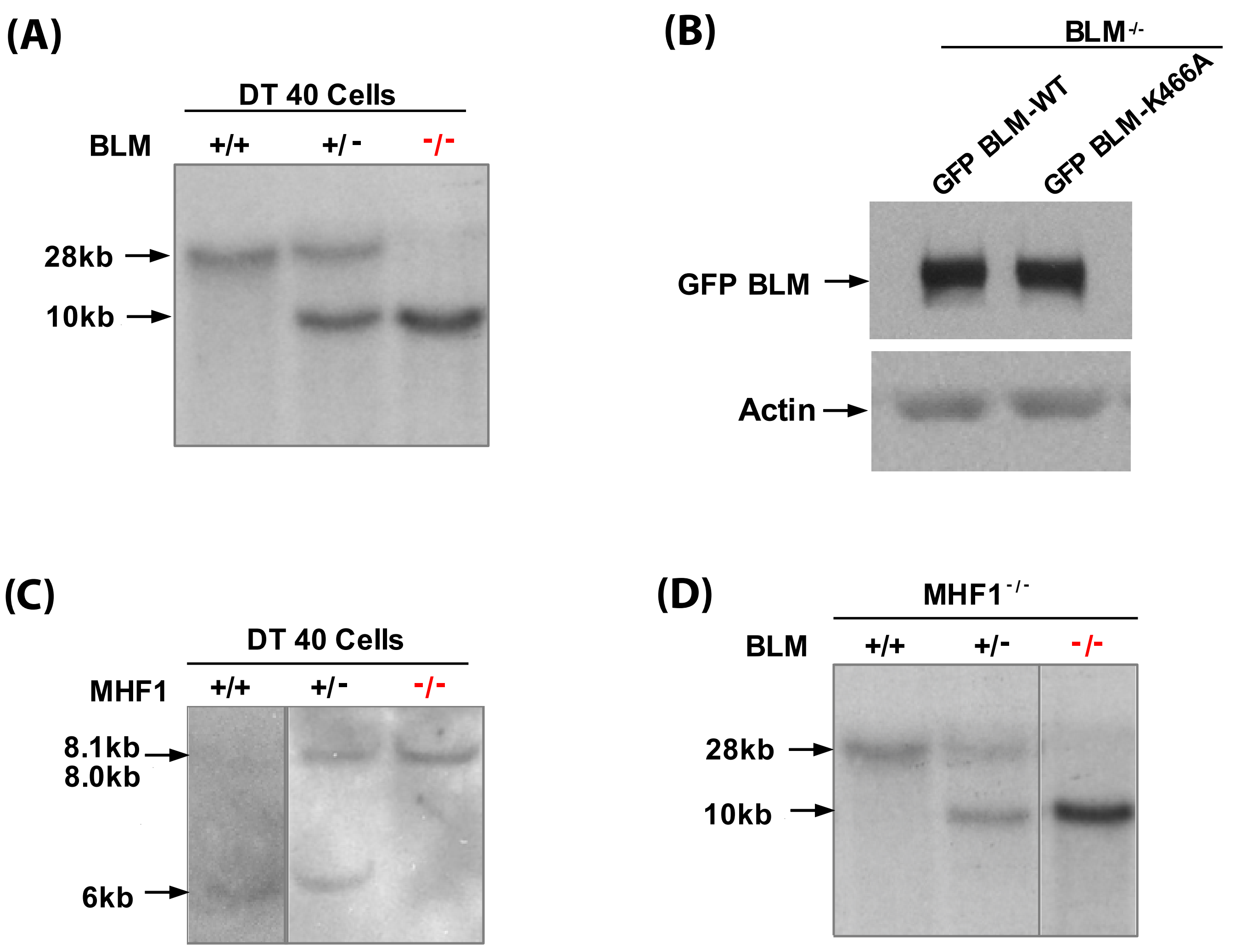

Legend: **(A)** Disruption of BLM gene in chicken DT40 cells. Southern blotting analysis of BLM-digested genomic DNA from the indicated genotypes.

(B) GFP-BLM wildtype(WT) or K466A mutant was introduced into BLM cells. The levels of their expression were analyzed by immunoblotting. Actin was used as a loading control.

(C) Same as (A), except MHF1 gene.

(D) Disruption of BLM gene in MHF1 DT40 cells. Southern blotting as performed as (A).

An MHF1 point mutant defective in DNA binding can promote FANCM recruitment to stalled forks as the wildtype protein

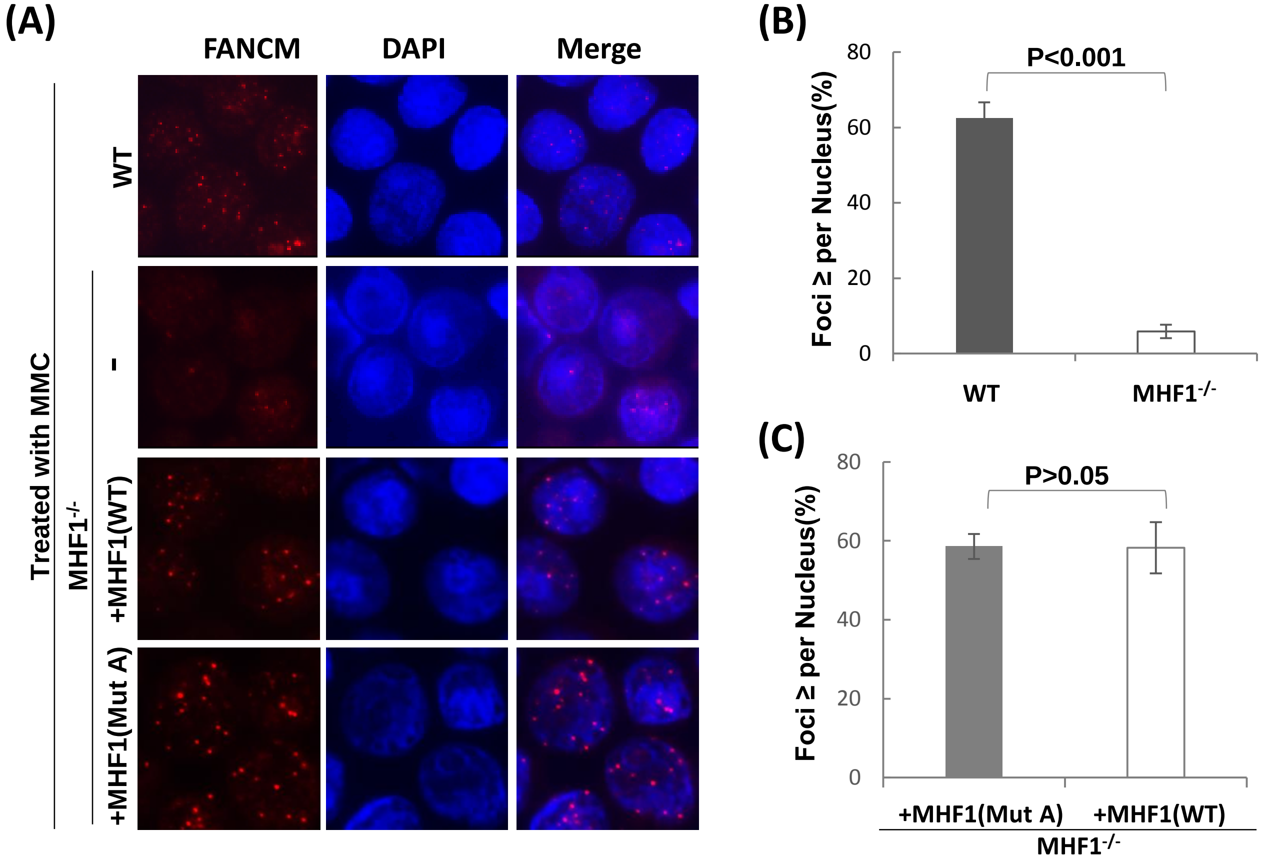

Legend. (**A**) Representative Immunofluorescence images, and (**B,C**) their quantifications, show that FANCM foci were reduced in *MHF1*$^{-/-}$ DT40 cells compared the wildtype cells; and this reduction was rescued by re-expression MHF1-wildtype or MHF1-mutant A protein. The MHF1-mutant A can stabilizes FANCM protein but is defective in helping FANCM to bind DNA. The fact that MHF1-mutant A can rescue FANCM foci formation suggests that the reduced FANCM foci formation in *MHF1*$^{-/-}$ cells is due to reduced FANCM stability in the absence of MHF, but not due to lack of contribution of DNA binding activity by MHF. Cells were treated with MMC (60ng/ml) for 18 hours before harvest and analysis of FANCM foci. (**B,C**) shows the mean and standard deviations (SD) of the percentage of cells containing ≥5 FANCM foci. *P* values between different cell lines are shown.

ATR but ATM is activated by replication stress in DT40 cells

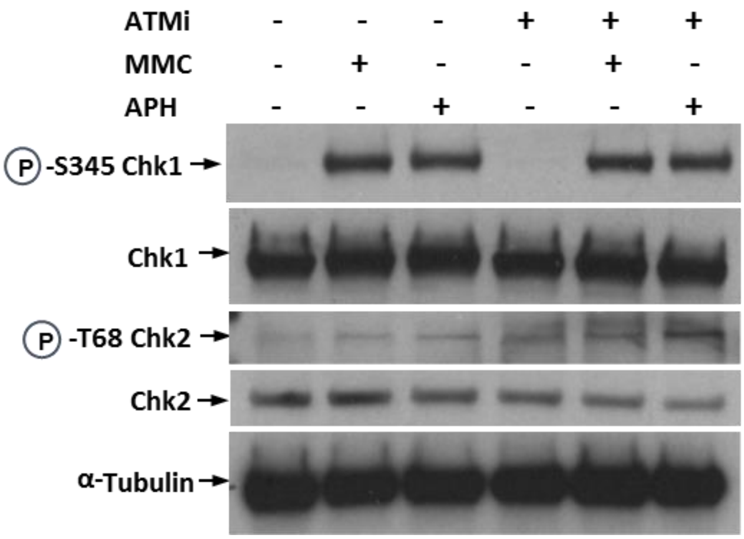

Legend. Immunoblotting images show that chk1, a major substrate of ATR, became hyperphosphorylated when DT40 cells were treated MMC or aphidicolin (APH) to induce replication stress. In contrast, a major substrate of ATM, chk2, did not show significant hyperphophorylation by the same treatment. The presence of a chemical ATM inhibitor, KU55933, did not alter phosphorylation statues of chk1 or chk2. Phosphorylation specific antibodies for chk1 (phosphorylated on S345) and chk2 (phosphorylated on T68) were used for detecting hyperphosphorylated chk1 and chk2, respectively. The presence or absence of the ATM inhibitor, MMC, and APH was indicated on the top. DT40 cells were untreated or pre-treated with ATM inhibitor (KU55933 at 10 µM) for 2h. They then either remained untreated, or were treated with MMC (50 ng/ml) or APH (5 µg/ml) in the presence or absence of KU55933(5 µM) for 18h.

ATR-dependent chk1 hyperphosphorylation is largely normal in *BLM*$^{-/-}$ and *RMI2*$^{-/-}$ DT40 cells

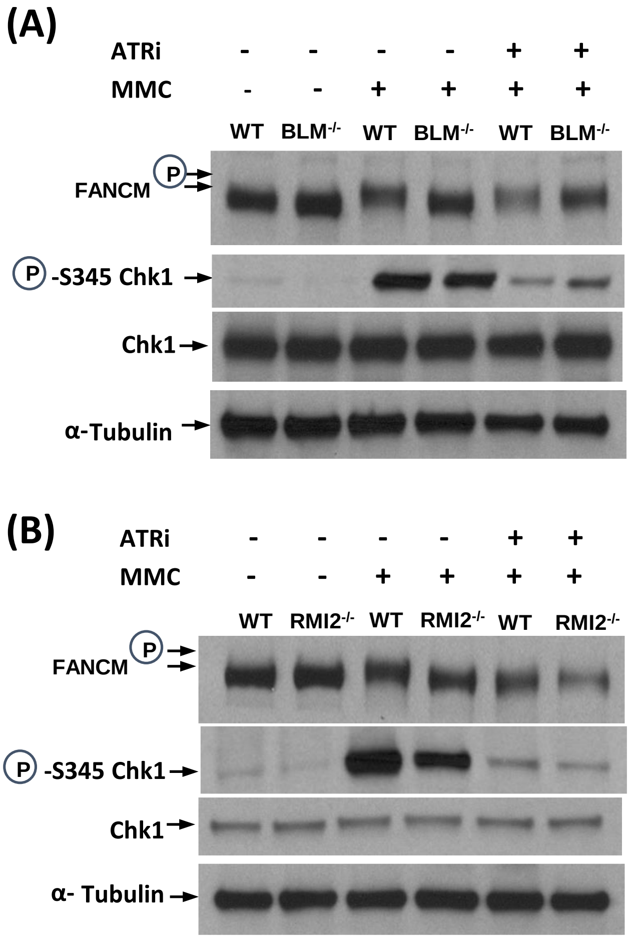

Legend. (A, B) Immunoblotting images show that the ATR-dependent chk1 hyperphosphorylation is largely normal in *BLM*$^{-/-}$ DT40 cells (**A**), or *RMI2*$^{-/-}$ DT40 cells (**B**), when compared to the wildtype cells. A phosphorylation-specific antibody for chk1 (phosphorylated on S345) was used for detecting hyperphosphorylated chk1. MMC was used to induce replication stress. Notice that MMC treatment strongly induced chk1 hyperphosphorylation in both wildtype and mutant DT40 cells; and this hyperphosphorylation was decreased by the ATR inhibitor. Cells were either untreated or pre-treated with an ATR inhibitor (VE821 at 0.6 μM) for 2h. They then either remained untreated, or were treated with MMC (50ng/ml) in the presence or absence of the ATR inhibitor (VE821 at 0.3 μM) for 18 hours as indicated on the top. α-Tubulin was used as loading control.

The chromatin-association of FANCM does not depend on the BLM complex

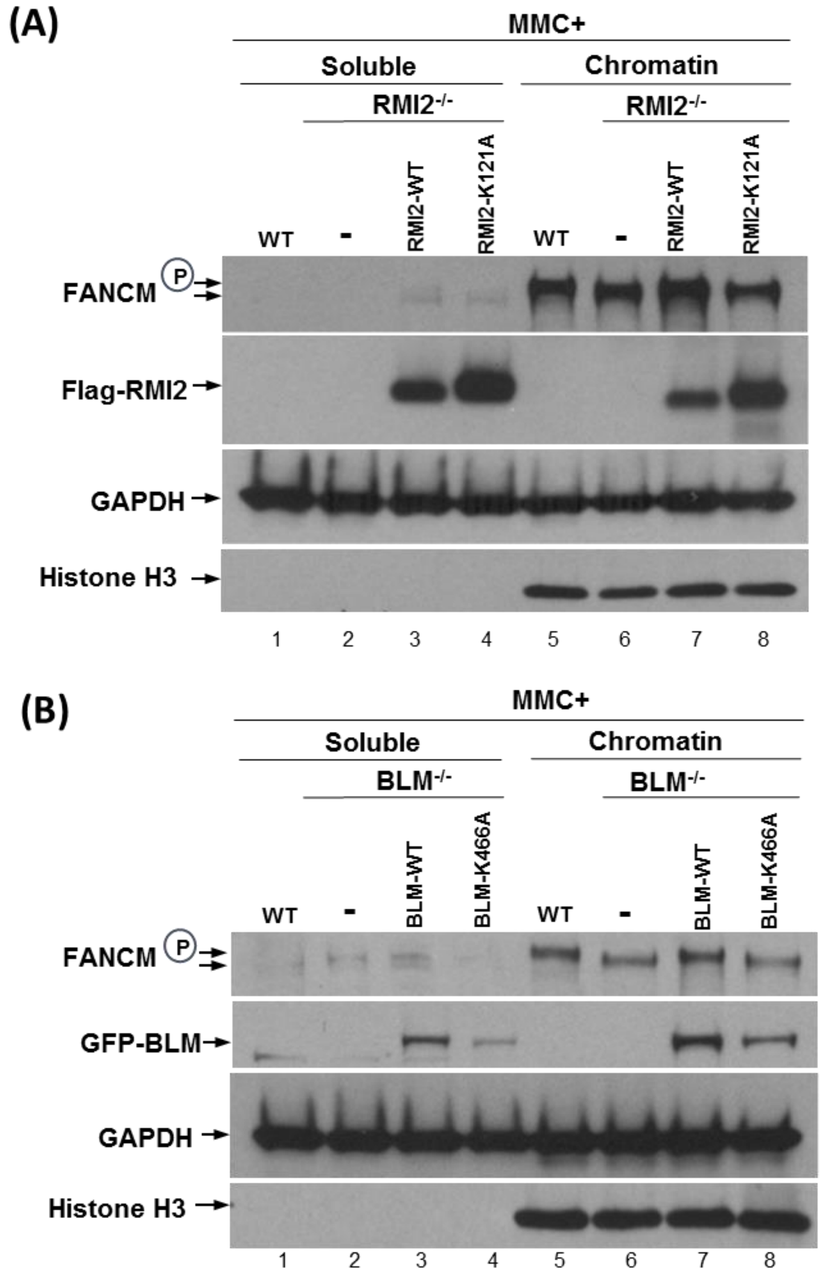

Legend. (A, B) Immunoblotting images show that the chromatin-association of FANCM is not altered in *RMI2*$^{-/-}$ (**A**) and *BLM*$^{-/-}$ DT40 cells (**B**), when compared to the wildtype cells. Histone H3 was used to mark the chromatin fractions. Cells were treated MMC to induce replication stress and FANCM hyperphosphorylation. Notice that most of the FANCM is always in the chromatin fraction, even though it is hyperphosphorylated in the wildtype cells (marked with P), but hypophosphorylated in the *BLM* and *RMI2* mutant cells, suggesting that FANCM chromatin association is not altered by its phosphorylation statues, or by mutations of the BLM complex. DT 40 cells were treated with MMC (60ng/ml) for 18 hours. GADPH were used as loading control**.** Also note that a large amount of Flag-RMI2 and GFP-BLM proteins were present in the soluble fractions, in contrast to FANCM which is mostly present in the chromatin fractions.
